# Vaso-Occlusive Pain and Menstruation in Sickle Cell Disease: A Focus Group Analysis

**DOI:** 10.1089/whr.2019.0008

**Published:** 2020-01-31

**Authors:** Melissa Day, Kemberlee Bonnet, David G. Schlundt, Michael DeBaun, Deva Sharma

**Affiliations:** ^1^ Vanderbilt-Meharry Center of Excellence in Sickle Cell Disease, Vanderbilt University Medical Center, Nashville, Tennessee.; ^2^ Department of Psychology, Vanderbilt University, Nashville, Tennessee.

**Keywords:** menstruation, sickle cell disease, vaso-occlusive pain

## Abstract

***Background:*** Acute vaso-occlusive pain, herein referred to as acute sickle cell disease (SCD) pain, associated with menstruation has received little attention. Key unanswered questions include how women differentiate acute SCD pain and menstrual cramps, and how both types of pain impact quality of life.

***Methods:*** Using inductive/deductive qualitative research methods, three focus groups were conducted to understand the patient experience of acute SCD pain associated with menstruation.

***Results:*** Fourteen women with SCD participated in our focus groups. Major themes were identified: (1) clinical sequelae of SCD surrounding menstruation, (2) coping with psychosocial challenges, (3) interpersonal difficulties and support systems, (4) impacts on quality of life, (5) impacts on emotional well-being, and (6) proposed solutions for health care systems.

***Conclusions:*** Women with SCD can distinguish acute SCD pain from menstrual cramps. Health care providers should become more familiar with acute SCD pain associated with menstruation and encourage a patient-centered dialogue to determine appropriate courses of action.

## Introduction

Acute vaso-occlusive pain (herein referred to as acute sickle cell disease [SCD] pain) is the primary reason for hospitalization in individuals with SCD^1,2^ and is associated with increased morbidity and mortality.^3^ The largest multicenter prospective cohort study of individuals with SCD (*n* = 3578) established that women have higher rates of acute SCD pain during their reproductive years.^4^ Some studies have attributed this difference to an association between acute vaso-occlusive pain episodes and menstruation.^5,6^

Modifiable risk factors associated with acute vaso-occlusive pain in women with SCD have not been fully characterized, and the SCD community has not widely recognized acute vaso-occlusive pain associated with menstruation as an entity. In a multicenter cross-sectional study (*n* = 221), almost one-third of women with SCD consistently reported experiencing acute SCD pain associated with their menstrual cycles,^7^ and dysmenorrhea and prolonged menstrual bleeding negatively impacted quality of life.^8^ However, we do not know the degree of impact that menstruation has on women with SCD with regard to interpersonal relationships, emotional well-being, interactions with health care systems, and coping strategies.

Using qualitative methodology, we sought to describe the experience of acute pain associated with menstruation in women with SCD, including health care utilization, symptoms experienced, coping strategies, and impacts on well-being. Our goal was to describe their life experiences and elicit suggestions for improving health services and outcomes.

## Methods

### Study design, inclusion/exclusion criteria, and recruitment

The moderator's guide was developed using input from health care providers who interact with women with SCD. The primary topics in the moderator guide were (1) describe your “sickle cell pain” and its relation to your menstrual period; (2) tell us the difference between your “sickle cell pain” and menstrual cramps; (3) discuss the frequency/consistency of “sickle cell pain” either during or directly before your menstrual period, and how the two are connected; (4) effect of “sickle cell pain” associated with menstruation on quality of life; (5) describe factors that worsen and alleviate “sickle cell pain” associated with menstruation; and (6) tell us what doctors need to know about treating this condition. The moderator asked open-ended questions using the guide, with specific probes and prompts for each question to encourage discussion.

Three focus groups consisting of women with SCD were conducted in October 2017 through January 2018. The focus group inclusion criteria were (1) female sex; (2) age 10–50 years; (3) a confirmed diagnosis of SCD; (4) currently having menstrual cycles, or requiring contraception due to severity of pain during menses; (5) history of experiencing acute vaso-occlusive pain either during or 1–2 days before their menstrual period; and (6) ability to speak and read English. Women who were not regularly menstruating due to pregnancy or menopause were excluded. This study was approved by the Institutional Review Board at Vanderbilt University.

We held the first two focus groups as part of the Sickle Cell Disease Association of America (SCDAA) conference in Atlanta, Georgia, in October 2017. Study flyers with focus group details were emailed to participants of the SCDAA national conference. We held the third focus group in Nashville, Tennessee, as part of a monthly Sickle Cell Foundation of Tennessee meeting. We screened participants for inclusion and exclusion criteria before the focus groups started.

### Procedures

Participants were consented upon arrival. A female moderator facilitated the discussion using the moderator guide. Each focus group began with an overview of the purpose of the focus group, acknowledgment of the presence of digital recording devices, reassurance of maintaining anonymity, and encouraging different viewpoints and experiences to be shared. At the end of the focus group, participants completed a short survey with demographic questions and questions about women's health issues. Each focus group lasted ∼2 hours, and participants were given a $50 gift card. Those assisting with the first focus group were debriefed to determine whether any modifications needed to be made for the second focus group iteration. The focus group discussions were digitally recorded and transcribed by a professional transcription service (rev.com).

### Data management and analysis

The Vanderbilt Qualitative Research Core managed and conducted the data analysis by following the consolidated criteria for reporting qualitative research guidelines,^9^ an evidence-based qualitative methodology. We developed and refined a hierarchical coding system by using the focus group guide and a preliminary review of the transcripts. Major categories included (1) sickle cell pain, (2) frequency/timing of sickle cell pain occurrence related to menstruation, (3) medications/methods for coping with menstrual pain, (4) quality of life, and (5) health system. These major categories were further divided from one to eight subcategories, with each subcategory having additional levels of hierarchical divisions. Definitions and rules were written to guide coding for each category.

Experienced qualitative coders first established reliability in using the coding system, then independently coded the transcripts. Discrepancies in coding were resolved to create a single coded transcript. Each statement could be assigned up to five codes. Transcripts were combined and sorted by code. Analysis consisted of interpreting the coded quotations and identifying higher order themes, using an iterative inductive-deductive approach. Deductively, theoretical contributions to the analysis included (1) clinician encounters with women with SCD, (2) social cognitive theory,^10,11^ and (3) a biopsychosocial framework.^12^ Inductively, the codes and themes from the focus groups were used to fill in conceptual framework details.

## Results

### Participant characteristics

Thirteen out of 14 women interested in the focus groups met inclusion criteria and participated. Four participants were in the first group, five were in the second group, and four were in the third group. The average age was 32.8 years (range: 19–46). Thirty-eight percent (5 of 13) had the Hemoglobin SS (HbSS) phenotype, 31% (4 of 13) had Hemoglobin SC (HbSC), and 31% (4 of 13) had Hemoglobin S-beta zero (HbSβ^0^) thalassemia thalassemia. All participants reported having at least a high school diploma.

### Qualitative data

**FIG. 1.**
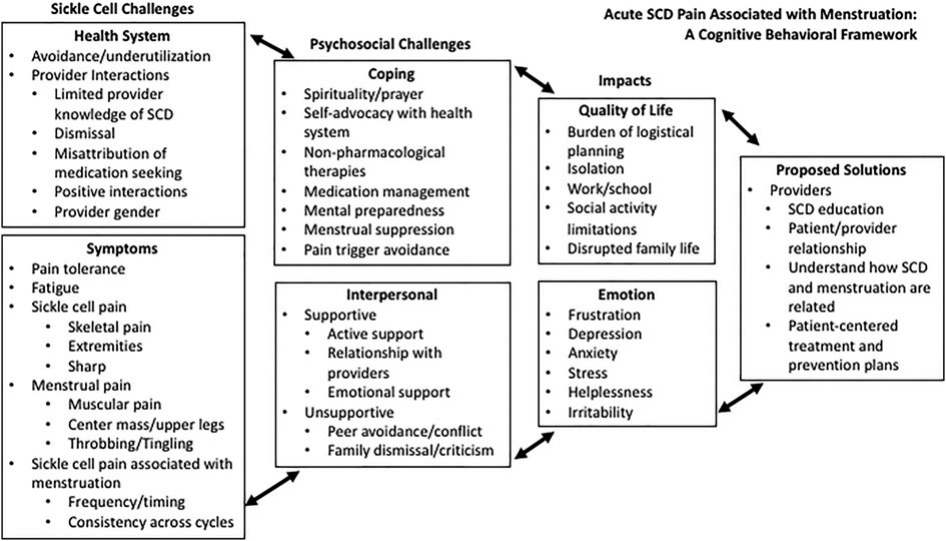
Acute SCD pain associated with menstruation: a cognitive behavioral framework. SCD, sickle cell disease.

Seven major themes were identified with regard to acute SCD pain associated with menstruation: (1) challenges in navigating the health system, (2) acute SCD pain outside of and associated with menstruation, (3) coping with psychosocial challenges, (4) interpersonal difficulties and support systems, (5) impacts on quality of life, (6) impacts on emotional well-being, and (7) proposed solutions for health care systems. Figure 1 organizes these themes. In the following sections, we discuss the major themes, enumerate subthemes, and include supporting quotations within tables 1–4.

**Table 1.** Quotations of Sickle Cell Disease Challenges Associated with Menstruation

Theme	Subtheme	Modifier	Quote
Navigating the health system			
	Avoidance/underutilization		“And to begin I start out with Motrin or Tylenol. I hate taking narcotics and so I will just try my best not to take it at all and the longer I wait, the worse it gets and I end up in the ER, which I hate going to, but yeah I try to deal with it at home with the low meds.”
	Provider interactions		
		Limited knowledge of SCD	“I kept flinching and he actually said … he said to me, ‘I don't know what this sickle cell thing is’ … I'm like, didn't you go to school?”
			“I've also found it difficult to explain to some of my primary care physicians that aren't familiar with sickle cell disease, truly what it feels like. When you're going through a crisis it can be … most last days, and you just continue with your day typically, or some days you're bedridden and just really can't move.”
		Dismissal	“When you were in peds, your doctors listened. You've been with them since like a baby, so they know you. Now that I'm an adult when I go into the hospital, which I said is every month, I have to fight and cry every single time just to get the medication that I need.”
		Misattribution of medication seeking	“So when I should be in here being treated… I'm under more stress and I'm already in pain, so that's an issue, extremely frustrating because they … We get the term ‘frequent fliers’ when they start seeing you so much. ‘Oh, she's just here for the drugs. She's just here for the drugs.’ And anybody who knows me know I hate taking medications. I will try to do everything I can before I have to take the medication.”
			“They're quick to give us the drugs…but then call us drug addicts.”
		Positive interactions	“Me and my doctor … that's exactly how it is. I call her my wife. I'm like, we sit and discuss everything. It's not her saying, ‘oh, you gotta do this, you have to do this.’ It's such and such is going on, let's look at alternatives and what we can do and let's discuss. We discuss it. Okay you want me to go on hydroxyurea for a year? I'm gonna give you six months. She's like, ‘how about?’ No, I was like, ‘I'll give you three months.’ She's like ‘how about nine?’ So I was like, ‘You know what? Let's meet in the middle. We could do six.’ It's a relationship you have to discuss.”
		Provider gender	“Cause a lot of doctors, no offense, are men, and they don't get it in the first place. They don't get cycles in the first place, they understand the science, but they don't get it….having the cycle, you know what I mean?”
Symptoms			
	Pain tolerance		“I have a high tolerance for pain now ‘cause when I was in labor I remember when I was in labor and you know how when you see people screaming and hollering? And I'm like when is it gonna be my turn, when I was in labor it was like, ‘Oh you're having another contraction.’, I said, ‘That's a contraction?’
			‘So they're like, ‘When your pain level gets to a certain level, we'll give you your epidural, so that you don't have to feel the whole pain of pushing out.’ And I was like, ‘Oh, okay.’ And I didn't ever get the epidural cause the level that I was waiting for never came because it was less … it wasn't as bad as having a sickle cell crisis.”
			“I remember when I had my first child I was in Walmart shopping for a humidifier pushing the cart and in labor, didn't know it, I thought it was just like back pain, so I was like just keep going. So by the time I got to the hospital I was like eight, nine centimeters ready to just push ‘cause I was like, ‘What is this?’ She was like, ‘Yeah, you're in labor.’”
	Fatigue		“I know it's coming, and I feel so tired and then there's like two days, it will be like almost like a total, like maybe three or four days where it's like oh, I'll go to work, go to sleep. Go to work, go to sleep like I don't want to do anything. I just can't, I mean besides the normal fatigue because there's always normal fatigue that I had where you got to push to get up, push to do everything and then it's just added. It's just added on, so it just makes everything worse.”
	Sickle cell pain		“I know the difference in my cycle pain and pain crisis. My sickle cell pain is sharp. It's never like some have aching or throbbing. I wish it was just aching and throbbing. Mine is just always stabbing, but when I'm on my cycle, that's when it just a throbbing, but when I'm in crisis, it's always a sharp pain, always, so that's the difference between mine.”
			“I know my sickle cell pain. My sickle cell pain feels different than any pain that I had like there's no
	Menstrual pain		“Menstrual pain is more muscular, whereas the sickle cell pain is more my joints and my back, my knees, you know my bones itself. So but my menstrual pain is more muscular, like my uterus is acting.”
	Acute SCD pain associated with menstruation		“I can distinguish from a cramp and my sickle cell pain, let me put it like that. So, I don't know if having sickle cell makes those cramps worse. Maybe the blood flow is a little heavier, but when you start your cycle and that's kind of when you're going through your cramps and stuff, they're bad, but I don't know how to describe it. It's not sickle cell pain bad let me put it like that. So, if sickle cell is ten, my menstrual cramps may be a four or five.”
			“And for me I can distinguish between the two pains, but my menstrual cycle directly correlates with my sickle cell. The losing of the blood would automatically put me into crisis. Automatically. So, immediately my back would hurt, my legs would hurt and I knew before my cycle started that I was gonna have my cycle by my sickle cell pre-warning.”
			“So, with my cycles, my crisis would be general mass. So it would be my stomach upper legs like where your cramp area would be, but then it would also be my lower back so my arms probably wouldn't hurt the whole time where in a non-menstrual one, my arms might be hurting or my legs might be hurting, my lower legs or something.”
			“The menstrual cycle does not compare to a sickle-cell crisis. It may induce a sickle-cell crisis but it doesn't compare to a sickle-cell crisis. Its pain is like oh, you don't like it but it's nothing compared to the crisis itself of a sickle-cell crisis.”
			“Sometimes I can't tell if I'm hurting because of my cycle or you know, it's just a regular pain crisis.”
			pain that compares to my sickle cell pain. That's why I know that even though I'm having cramps, I'm having a crisis in my legs, and I'm, my legs and my arms and stuff or hurting in this crisis pain and not just pain. So my sickle-cell pain is just different than any kind of pain I have, whether it's the pain that's on my side, whether it's my menstrual pain, that's it.”
	Frequency of acute SCD pain associated with menstruation		“As long as I have a period, I have sickle cell pain and that's why my doctor was like we're shutting this down, you ain't about to have no period, like because there's too many complications that are going on every time your period comes.”

SCD, sickle cell disease.

#### Challenges in navigating the health system

Similar to the general SCD population, participants in our study frequently avoid utilizing health care services, due to previous negative provider interactions and avoidance of controlled substances (Table 1). Participants reported that their providers had limited knowledge of SCD and how to appropriately treat their condition in both the acute (inpatient/emergency department) and nonacute (primary care, obstetrics/gynecology (OB/GYN), and hematology outpatient clinic) settings.

Particularly in acute settings, women stated their concerns about acute SCD pain associated with menstruation were dismissed and not taken seriously. One described that the transition from pediatric to adult care made getting the medications she needed more difficult. Many discussed being labeled as medication seekers when they go to the emergency department and request specific medications they know will appropriately treat their pain.

Specific to women's health concerns, our participants voiced that male providers may not fully understand menstruation and associated pain, and how to acutely treat individuals with SCD. However, some women gave positive reviews of interactions with certain health care providers, with most referencing a female provider who created a sense of partnership, with patients being able to express concerns and integrate them into their health care decision making.

#### Acute SCD pain, menstrual pain, and acute SCD pain associated with menstruation

Women with SCD described the symptoms they experience outside of and in association with menstruation, including menstrual cramps and acute SCD pain (Table 1). Participants reported that they experience high pain tolerance compared with women without SCD. They described that menstrual cramps were much lower in pain severity than acute SCD pain. Other women spoke about how an acute SCD pain episode is worse in severity than the pain of childbirth.

Women compared their acute SCD pain with menstrual pain, describing their acute SCD pain as a sharp skeletal pain in their extremities, whereas menstrual pain felt more muscular and throbbing/tingly, occurring in their “central mass” and upper legs. Participants stated they could distinguish their menstrual pain from acute SCD pain. Women made it clear in the discussion that acute SCD pain was more severe than menstrual cramps.

Women then spoke about acute SCD pain associated with menstruation, stating that their menstrual period would directly put them into an acute SCD pain event. Some women spoke about differences in location of acute SCD pain occurring during and outside of menstruation, with acute SCD pain during menstruation occurring in their “general mass.” One woman said she could not distinguish between her acute SCD pain and menstrual cramps.

Women discussed the frequency and timing of pain relative to menstruation and how consistent this pain occurs across cycles. The majority of women described that their acute SCD pain occurred with every period, prompting health care providers to seek therapies to suppress menstruation.

#### Coping with psychosocial challenges

Women described multiple methods to cope with acute SCD pain associated with menstruation (Table 2). Some women discussed the importance of faith and relying on spirituality, prayer, and their relationship with god to get them through the pain of menstruation.

**Table 2.** Quotations of Psychosocial Challenges in Sickle Cell Disease Associated with Menstruation

Theme	Subtheme	Modifier	Quote
Coping strategies			
	Spirituality/prayer		“Even though my job is stressful, I've come just a point in my life where I'm in a different, and you know talk about coping spiritual, that is definitely a coping mechanism because it's like it comes to this point where you get on this level with God where when I say Lord I just need you right now, I need you to take this pain away and He does it…I remember Him just saying I got that, and I gave it to him and I actually let it go.”
	Self-advocacy with health systems		“Ask your doctor, tell your doctor, demand that your doctor understands your disease and gives you a treatment plan for your disease. And that's what we did, we became friends and she treated me as such and she put a plan in place and when I would have crises during the pregnancy, I would just go to the hospital, they knew exactly what to do.”
	Nonpharmacological therapies (heat, holistic approaches, and therapy)		“And it depends on what's going on because I try to take the holistic approach…with Epsom salt and bubble bath, and I sit in that as long as I can… or I would go to a spa and get a deep tissue massage and that would help a lot.”
			“Heat makes everything better.”
			“So I know for me, you know, my therapist says that one tool, that kind of is my go to, like, all right. You know I'm starting to feel this, I'm starting to feel that. This is about to happen. This is about to happen. And my mind is going here and so that's one of my tools that I use. So like I said I've talked to her about everything, from family to menstrual to projects, to everything. So that's one of my go to tools but that's not going to be everybody's go to tool. You know, we're all different, we all have different experiences, so you know, just being able to get that round table of different ages, different ethnicities, different life walks of just you know hearing everybody's story.”
	Medication management		“If I can catch it early enough and I recognize that, that's what's happening I can either take a high dose of ibuprofen like a 800 ibuprofen, maybe one or two of those could start to dull the pain, but if I don't feel that the pain is being dulled in like three hours or something like that, I know that it's more than that, Imma need an opioid or some type of higher dose.”
			“The regular stuff that people can get away with taking during their cycle does not work for me, at all. So I have to take Dilaudid or … they just put me on tramadol, so now I'm on three opioids all together and that's not including ibuprofen and stuff for breakthrough pain.”
			“I'm probably bad for this, I practically punish myself, I hate taking really strong pain pills so I will deal with the ibuprofen's and I'll rotate between the higher ibuprofen's and Tylenol's and stuff and I'll go as long as two or three days before I go as far as taking an opioid, yeah. ‘Cause I just don't like the … I don't like nausea, throwing up, all the other stuff.”
	Menstrual suppression		“So for me it was, my doctor was like oh no, we shut this thing down right now…so I'm on Depo. So every three months I had a shot but they gave me so much that they literally shut my period down so I would go without a period.”
			“Once I started birth control it regulated my periods so I would still get them every month. Sometimes I would skip months or take my birth control continuously so I could skip a period, just to avoid having that crisis pain.”
	Pain trigger avoidance		“It is always the stress that brings on those sickle-cell crises. It's, my biggest trigger is stress, so if my anxiety is kicking up when the hormones of you know the menstrual is coming.”
Interpersonal relationships			
	Supportive interactions		
		Active support	“My best friend, he's okay with it, he's always giving me either apple cider, cocoa, he's always giving me something warm.”
			“I typically don't even go to the hospital anymore without someone with me so they can explain they can, even when I can't talk, I need them to kind of be that spokesperson for me. I am an advocate for myself, don't get me wrong but when I'm in the middle of a 10 crisis, and most of the time I can't even articulate it to the bedside person whether it's the nurse or the doctor. And a lot of times when they're not listening, I just start crying and so the person with me knows to jump in and say ‘Okay, she needs morphine and phenergan so that it doesn't make her sick, get her a garbage can, she needs an IV like now’, you know those types of things and that's happened on I can't begin to have a count.”
		Relationship with providers	“The good thing about what happened to me was I got a pain management doctor who was a woman who understood both, and she was like momma to me. She wound up kind of talking to me about my cycle before I even spoke to her about it. So she … she be telling me stuff that I didn't even think about.”
		Emotional support	“My best friend's wedding was coming on and she said I want you to be a bridesmaid and I didn't want to commit because I didn't want to disappoint her. I was like because there's a off-chance that I may not be able to make it if I have to be hospitalized and I don't want to. She's like even if we have to have you on video from the hospital, you're going to be a part of my wedding. So and that made me encourage me to. Because I knew, like my period was starting to come more regular and the date she had her wedding set was when my period was most likely to come and I said I don't know, you know. And I'm like I don't want to not be there for you and she was like I don't care. You know she listed me as her bridesmaid, she was like you know if we have to have the video camera up there and you have your bouquet in your hand in the hospital, you're going to be my bridesmaid.”
	Unsupportive interactions		
		Peer avoidance/conflict	“I know my friends know because they can look at me and they're like, ‘uh, you know what? We gonna catch you later. You ain't even got a smile on your face.’”
		Family dismissal/criticism	“My sister be like, ‘Well are you taking your meds? Are you doing what the doctor said?’ And I'm like, yes. ‘Then you shouldn't be sick.’ And I'm like that's not always the case. ‘Cause my family be like, ‘well if you would take your meds like you should, you wouldn't be sick’ … so even when I get sick and go to the hospital, I will stop telling them because they'll be like ‘if you were doing right, you wouldn't be in the hospital.’ I'm like I'm doing everything, its just not working.”
			“But again I had issues with the family and oh, you ain't got bipolar and we're just going to pray that away. And it's just like, yeah, sometimes I got to be on medicine…I got pulled to the front, I got prayed over. I got cast out that demon and it's just like I have to do what I have to do to get through life.”

To cope with these challenges, many women reported how self-advocacy within the health system worked for them, demanding that their doctor understand their disease.

Women reported using both nonpharmacologic and pharmacologic methods to ease their acute SCD pain associated with menstruation. For nonpharmacologic interventions, many women reported that heat (heating pads, hot showers, and hot tea) and holistic approaches helped alleviate their pain. Women also discussed seeking a therapist to help them manage pain from menstruation and other stressors.

For pharmacologic interventions, women rely on ibuprofen or acetaminophen, only resorting to opioids when ibuprofen or acetaminophen does not alleviate their pain. Some women reported that they start with an opioid, the only medication that controls their pain.

Owing to the consistency of acute SCD pain across menstrual cycles that some women reported, they have been placed on hormonal contraception. Women described that their doctor understood the necessity of menstrual suppression to control acute SCD pain.

Women briefly discussed that around the time of menstruation, they actively try to avoid known pain triggers, such as cold environments and stress, and mentally prepare themselves for pain when their menstrual periods are about to start, a time of high anxiety.

#### Interpersonal difficulties and supports

Women discussed both supportive and unsupportive actions from friends and family about their acute SCD pain associated with menstruation (Table 2). Concerning emotional support, one participant described how her friend supported her even when she would likely be missing her wedding. Women also spoke about how their relationship with their providers gave them a sense of support.

Regarding unsupportive actions, women discussed how family members criticized them by saying they were not doing everything they could to control the pain, including not taking their medications as prescribed or following the doctor's orders. One woman described how her family did not accept that she needed to be on medicine and suggested that she “pray that away.”

#### Impacts on quality of life

Women discussed how acute SCD pain associated with menstruation impacted their quality of life, including the burden of logistical planning, isolation, missing and leaving work/school early, and social activity limitations (Table 3).

**Table 3.** Quotations on Impacts from Acute Sickle Cell Disease Pain Associated with Menstruation

Theme	Subtheme	Quote
Quality of life		
	Burden of logistical planning	“For me, it affects my life because I have to do a lot of planning, so I have to get people on place, like I said I have kids and so I have to do a lot of planning and I think that's sickle cell in general, especially if you work and you know you have, I have to always plan out and think ahead of, okay, what's going to happen. If it's coming, I got to have somebody on call.”
	Isolation	“So when I was on my cycle, I would keep to myself. I would try to stay quiet because you could come up and say something and the littlest thing will set me off and then I done and blew up on somebody for no reason, and they're like, ‘what did I do?’ You didn't do nothing. You just existing and I happen to be a sickle cell patient on my cycle.”
	Work/school	“In July when I said I had the really bad cycle … I got fired because I had to drive myself from South Jersey back up to North Jersey and go to the hospital because of my period because I was feeling so weak. I didn't have no energy. I was in so much pain, it was like okay I had no choice but to leave and I drove straight back up to North Jersey where my doctor and my hospital was. So I am not currently working now because of it.”
		“I'd literally call my dad…I have literally called him and made up a story saying, ‘Can you come get me, I just need to run home real quick,’ and go home and take something and literally have to lay down for 10 minutes and then go check back in the school when I was on my cycle. That was every time I was on my cycle and when it like happened in school, you know most girls just keep their little supplies with them, which I did, but when it first come on, I would have to go home and regroup and then go back to school and nobody knew like because I would make up a reason why I would have to go home. They didn't notice because of my cycle was coming on and I was not able to function when it first came on.”
	Social activity limitations	“My best friend graduated from law school and right before I booked the ticket and everything to get down there to see her walk across the stage and I got sick right before. So I missed her entire graduation and even though she made me feel better about it, like she was like oh, it was hot and you probably wouldn't have enjoyed it anyway because you know it was so crowded and it was such a hot day, I'm like but I still want to be there though. I felt so bad in that, you know, this is your best friend, this is her dream to be a lawyer and I couldn't be there to support her in this moment.”
		“So it's been plenty of time to where like I've had like big responsibility that I couldn't even fulfill because my body just shuts down or go into crisis or whatever so I had to deal with that in disappointing people. I felt like I can't commit because I'm going to disappoint them if something happens to me.”
		“And then try to explain to them well mommy don't feel like doing it so I can't be there for this and that, it just, it gets depressing at times.”
Emotion		
	Frustration	“It's frustrating as a patient because it's like no matter what you do, you still hurt. It's frustrating for doctors, so they're frustrated, and then you're frustrated, sometimes that doesn't mix. And then, I'm under the stress of transitioning from peds to adults. I know for a teenager, that's stressful for young adults, because it's a whole new world switching from pediatrics to adults.”
	Depression	“Because one time I was in the hospital for like two weeks and I was still in pain and the doctor was like we can't give you more pain medicine and stuff and I got deeply depressed and I told them I said I need to see someone because I'm ‘bout to go crazy up in here and I really was. I was on the verge of suicide. I was like I can't keep going through this. This is just too much, I can't.”
		“And the doctor wasn't listening to me cause I'm like there's, something gotta be wrong … I been here 13 days and my pain is still at a 10 well over a 10, but they only have a 10 scale and you all can't give the meds, you know I'm not here seeking drugs, I am in pain. And I was ready to, I just wanted to end it and I told the nurse, please send me a social worker or someone in here I could talk to because I am very depressed. Through all the pain and tears, I'm depressed. I had cried so much, I couldn't even cry no more. And I was just in so much pain, so yeah I've been at that point.”
	Anxiety/stress	“I know for me stress is my biggest [stressor]. So I know when the hormonal stuff starts happening with my period, that stresses me out, my anxiety goes up and then I start freaking out like, oh man, here it comes…then I start freaking out and I'm like oh man, my sickle cell is about to come now too, so the whole thing that's going on in my mind alone, it's starting to make my body react to what's going on.”
	Helplessness	“I get depressed because I feel helpless, like I can't help the kids do nothing, I'm just and oh my gosh, it is depressing, for me it is ‘cause I feel like I can't do nothing.”
	Irritability	“It's irritating. Like it's one thing to be like on a sickle cell crisis but when you are on your menstrual and you're in pain, it's the most irritating thing because you're moody, you're aggravated, you don't want to be bothered and then you also like are in pain. It's just like I'm not nice. I'm this completely different person. It's very aggravating.”

Women reported that they can get consumed with the amount of contingency planning around menstruation, especially involving their children. Participants stated they would isolate themselves from family/friends to avoid upsetting someone from the stress of menstruating.

Women described how they have not only missed work due to acute SCD pain associated with menstruation, but also were fired from their job or missed school due to the significant amount of pain they experienced.

In terms of social activity limitations, one participant described that she had to miss her friend's law school graduation due to having acute SCD pain associated with menstruation. Women stated that their family time was disrupted due to having acute SCD pain associated with menstruation, including missing out on their children's accomplishments.

#### Impacts on emotional well-being

Participants described the emotional impacts they experienced due to acute SCD pain associated with menstruation (Table 3). These included frustration, depression, anxiety, stress, helplessness, and irritability.

Women described that stress and anxiety are not only triggers for acute SCD pain, but occur when anticipating their menstrual periods, leading them to have an acute SCD pain episode. A participant experienced severe depression when hospitalized for acute SCD pain associated with menstruation, prompting her to ask for a social worker or therapist. One woman described that her irritability stemmed from having both menstrual and sickle cell pain occurring during her periods.

#### Proposed solutions for the health care system

Participants recommended that their doctors seek education about SCD, encourage a patient–provider partnership, understand that SCD and menstruation are related and cannot be separated, provide patient-centered treatment and prevention plans, and discuss women's health issues such as pregnancy, fertility, and genetics (Table 4).

**Table 4.** Quotations for Proposed Solutions to the Health Care System

Subtheme	Quote
SCD education	“Same thing with OB's. My OB… I had to educate my OB, get her up to speed, I had to tell her, first off I told her that I was Beta Thal and she went and did some research and by the time she did her research I was pregnant, so even during my pregnancy when I would have crises, she would put a plan in place for me and had it at the hospital. I would just go to the ER, they pull up my file and they already have a treatment plan ready for me. And that's how she treated me and that's my expectation from any doctor.”
Patient–provider relationship	“I think more people need to talk about it during transition, like during the transition period of you come into you know, from peds to adults, I think that needs to be in the conversation. They were just like bah. So now that we have the programs and the protocols and the transition, you know okay, let's add this menstrual and you know proactive thing we can do, you know when it comes and things like that and have that plan of action and have that you know.”
	“I think, yeah, for this season in my life because I think somebody said yesterday your kind of in a transition from like high school to college then college to like adulthood. You know you kind of going into family mode, life mode and like we need a plan of action for that too, like, because if I'm at this point, I have sickle-cell and dealing with the menstrual but then now talking about family, talking about kids, like yeah, there needs to be people in place that will walk families through this process. Especially when dealing with like menstrual cycles, children, all of that stuff. We want more support from our doctors. We want more support from our counselors. We want more support from the people who are taking care of us on a daily basis. We want more support. We want people to see us as individuals and not as a number. That's what we want.”
Understand how SCD and menstruation are related	“One thing that doctors do need to understand that sometimes the two play a part, that sickle cell is that, our menstrual isn't just separated from both. They need to understand that our periods can cause us to have crisis attacks or our crisis attacks can make our periods worse. Some doctors don't understand that because they really don't see a lot of sickle cell patients and that aspect, especially as women, that aspect we don't really discuss.”
	“I don't know why it happens, I'm not, I mean because I know sickle-cell is the blood, right. So blood is everywhere, it's in every part of body, right. But why do I go into a crisis mode because of my menstrual cycle? And is not even here yet. So I know we go through the ovulation and like I can, like I know, which side, like it's weird. I know, which side my it drops from, whatever that mean.”
Patient-centered treatment and prevention plans	“Be more proactive and have a plan like, have a plan of action. What are we going to do so that this is prevented and you know, have more preventative measures than reactive measures.”
	“I want them to know like everybody else said, when we go, before the menstrual comes up, we're already feeling it. What can you do to help us you know, with this, so that we don't end up, by the time we get up here, we don't end up in crisis? That's what I want them to know.”
	“But let's figure out alternative ways because I hate medicine. And sometimes I do that anyway, like with my folic acid, I know green vegetables are high in folic acid. So instead of taking that, I take something else and for pain medicine, there's something else I used to eat that helped with pain. So instead of medicine, let's figure out another way because I hate taking medicine.”
Discussion of women's health issues in SCD	“Sex, like I ain't never known sex could send you to the … you could have a crisis attack with sex. Found that out the hard way. Things like that aren't discussed because I don't know whether they feel like it's a taboo, or they don't trust their doctors enough to discuss that, but those are the things that need to be discussed because if you can't trust your doctor and talk to your doctor about that, you need a new doctor. That's the purpose of having that doctor is to have those conversations; the hard ones that you can't have with maybe family and friends, so they can give you advice and you guys can work it though.”
	“We don't talk about pregnancy. We don't talk about fertility, and genetics, and what's going on period wise, and things like that.”
	“Tell me what I can do, not what I can't do.”

Women were very interested in having their providers treat *and* prevent their acute SCD pain associated with menstruation. They wanted the emphasis to shift from medications to alternative methods to avoid highly potent medications.

Participants also reported that acute pain associated with menstruation and other reproductive health topics are not commonly discussed during transition from pediatric to adult care, but should be.

Women addressed being told continuously about limitations to quality of life from SCD, instead of being empowered and told what they are able to do. One woman was told not to become pregnant due to potential complications.

Participants also requested that their physicians seek to understand the underlying biology of acute SCD pain associated with menstruation. Women want to know what is causing their pain so they can plan accordingly to have a better quality of life.

## Discussion

Acute vaso-occlusive pain episodes are the most frequent complication of SCD. Despite this, relatively few studies have addressed whether acute vaso-occlusive pain is temporally associated with menstruation. In this study, we found that most women reported experiencing acute SCD pain and menstrual cramps with every menstrual period. Through our focus group analysis, we have captured that the experiences of women with SCD have led to difficulties navigating the health care system, particularly with male providers, and impacts on quality of life, interpersonal relationships, and emotional well-being. These problems have led women to find coping strategies and supportive relationships, and propose solutions for the health care system.

This is the first qualitative study to describe acute SCD pain associated with menstruation from the perspective of women with SCD. Previous studies have reached conflicting conclusions regarding whether an association between acute SCD pain and menstruation exists and, therefore, have not explored this topic further.^13–16^ In a recent multicenter cross-sectional study, almost one-third of surveyed women with SCD (*n* = 221) consistently experience recurrent acute SCD pain 0–7 days before or during menstruation.^7^ Most women in our focus groups confirmed that acute SCD pain occurs with every menstrual period and they can distinguish acute SCD pain from menstrual cramps. Our focus group discussions reaffirm that acute SCD pain associated with menstruation is a real patient experience for women with SCD and should be rigorously studied.

A unique attribute of our study is the elicitation on how to improve the health care system, which is currently lacking in the literature. This is the first study to our knowledge that utilizes qualitative methods to explore the experience with acute vaso-occlusive pain and the temporal relationship with menstruation in SCD.

Women voiced multiple experiences within the health care system that should prompt improvement in their management, especially regarding acute SCD pain associated with menstruation. Interactions with providers who lack knowledge about SCD and dismiss women's health concerns discouraged women from seeking appropriate care. These negative relations tended to involve male providers and the misattribution of medication seeking. When asked what providers need to know to better care for women and acute SCD pain associated with menstruation, women stated that providers needed to be educated on the multiple aspects of SCD and create a collaborative partnership with their patients. Women also emphasized the need to develop preventative strategies for acute SCD pain associated with menstruation, encouraging providers to understand how the two are related. Therefore, our findings suggest that health care providers stay current in the management of SCD.

This study had some limitations. First, these focus groups may have disproportionately appealed to women with more severe disease to come and speak about their experiences with acute SCD pain associated with menstruation. However, by conducting these groups at the SCDAA national community-based organization annual conference, we recruited a heterogeneous population of women across the country who shared their unique experiences with SCD. In addition, the problems in health care systems and proposed solutions raised by participants will likely positively influence future care for all women with SCD. Second, the focus groups were small (*n* = 13). We might have learned about additional experiences, problems, and suggestions had we talked to a larger number of women.

Women with SCD can distinguish acute SCD pain from menstrual cramps, with most of our participants experiencing acute SCD pain with every menstrual period that has negatively impacted their emotional and interpersonal well-being and interactions with health care systems. These impacts on quality of life should prompt increased awareness and education among health care providers concerning the management of SCD and acute pain associated with menstruation, and encourage a patient-centered dialogue to determine preventative and therapeutic courses of action.

## Abbreviations Used

SCD: sickle cell disease
SCDAA: Sickle Cell Disease Association of America

## References

[1] Kauf TL, Coates TD, Huazhi L, Mody-Patel N, Hartzema AG The cost of health care for children and adults with sickle cell disease. Am J Hematol 2009;84:323–327 19358302 10.1002/ajh.21408

[2] Ballas SK The cost of health care for patients with sickle cell disease. Am J Hematol 2009;84:320–322 19415728 10.1002/ajh.21443

[3] Platt OS, Brambilla DJ, Rosse WF, et al. Mortality in sickle cell disease. Life expectancy and risk factors for early death. N Engl J Med 1994;330:1639–1644 7993409 10.1056/NEJM199406093302303

[4] Platt OS, Thorington BD, Brambilla DJ, et al. Pain in sickle cell disease. Rates and risk factors. N Engl J Med 1991;325:11–16 1710777 10.1056/NEJM199107043250103

[5] Yoong WC, Tuck SM Menstrual pattern in women with sickle cell anaemia and its association with sickling crises. J Obstet Gynaecol 2002;22:399–401 12521464 10.1080/01443610220141362

[6] Samuels-Reid J, Scott RB Painful crises and menstruation in sickle cell disease. South Med J 1985;78:384–385 3983658 10.1097/00007611-198504000-00007

[7] Sharma D, Day ME, Stimpson SJ, et al. Acute vaso-occlusive pain is temporally associated with the onset of menstruation in a subset of women with sickle cell disease. J Womens Health 2019;28:162–169 10.1089/jwh.2018.7147 30648915

[8] Day ME, Stimpson SJ, Rodeghier M, et al. Contraception use and the impact of menstrual characteristics on quality of life in adolescent and adult women with sickle cell disease. South Med J 2019;112:174–179 30830232 10.14423/SMJ.0000000000000949

[9] Tong A, Sainsbury P, Craig J Consolidated criteria for reporting qualitative research (COREQ): A 32-item checklist for interviews and focus groups. Int J Qual Health Care 2007;19:349–357 17872937 10.1093/intqhc/mzm042

[10] Bandura A Human agency in social cognitive theory. Am Psychol 1989;44:1175–1184 2782727 10.1037/0003-066x.44.9.1175

[11] Mischel W, Shoda Y A cognitive-affective system theory of personality: Reconceptualizing situations, dispositions, dynamics, and invariance in personality structure. Psychol Rev 1995;102:246–268 7740090 10.1037/0033-295x.102.2.246

[12] Engel GL The clinical application of the biopsychosocial model. Am J Psychiatry 1980;137:535–544 7369396 10.1176/ajp.137.5.535

[13] Isaacs WA, Effiong CE, Ayeni O Steroid treatment in the prevention of painful episodes in sickle-cell disease. Lancet 1972;1:570–571 4110053 10.1016/s0140-6736(72)90359-5

[14] Serjeant GR, Ceulaer CD, Lethbridge R, Morris J, Sinhai A, Thomas PW The painful crisis of homozygous sickle cell disease: Clinical features. Br J Haematol 1994;87:586–591 7993801 10.1111/j.1365-2141.1994.tb08317.x

[15] Westerman MP, Bailey K, Freels S, Schlegel R, Williamson P Assessment of painful episode frequency in sickle-cell disease. Am J Hematol 1997;54:183–188 9067495 10.1002/(sici)1096-8652(199703)54:3<183::aid-ajh2>3.0.co;2-s

[16] de Abood M, de Castillo Z, Guerrero F, Espino M, Austin KL Effect of Depo-Provera or Microgynon on the painful crises of sickle cell anemia patients. Contraception 1997;56:313–316 9437560 10.1016/s0010-7824(97)00156-x

